# Unveiling drug resistance pathways in high-grade serous ovarian cancer(HGSOC): recent advances and future perspectives

**DOI:** 10.3389/fimmu.2025.1556377

**Published:** 2025-04-30

**Authors:** Ruiting Fu, Ruiyue Hu, Wenting Li, Xifang Lv, Hanwei Zhao, Fuxia Li

**Affiliations:** ^1^ Gynaecology department of The First Affiliated Hospital, Shihezi University, Shihezi, Xinjiang, China; ^2^ Gynaecology department, The People’s Hospital, Shihezi University, Shihezi, Xinjiang, China; ^3^ Peripheral vascular disease department of The First Affiliated Hospital, Heilongjiang University of Traditional Chinese Medicine, Harbin, Heilongjiang, China

**Keywords:** high-grade serous ovarian carcinoma (HGSOC), drug resistance mechanisms, molecular markers, personalized treatment, tumor microenvironment

## Abstract

High-Grade Serous Ovarian Carcinoma (HGSOC) represents the most prevalent and lethal subtype of ovarian cancer, with approximately 225,000 new cases reported globally each year and a five-year survival rate of merely 49.1%. The clinical management of HGSOC encounters substantial challenges, primarily attributable to its intricate drug resistance mechanisms, which involve multiple biological processes, including tumor cell heterogeneity, microenvironment remodeling, gene mutations, and drug efflux. This study systematically reviews the most recent advancements in HGSOC drug resistance research, concentrating on the molecular biological foundations of resistance mechanisms, innovative detection strategies, and potential therapeutic approaches. The research indicates that HGSOC drug resistance constitutes a complex process characterized by multifactorial interactions, involving aberrant cell signaling pathways, dynamic alterations in the tumor microenvironment, and specific expressions of molecular markers. In this review, we systematically analyzed and investigated the intricate biological behaviors associated with HGSOC drug resistance, which not only enhances the understanding of disease progression but also provides essential theoretical foundations for the development of more precise and effective targeted therapies. This review firstly illustrated the detailed drug resistance cellular and molecular mechanisms underlying HGSOC chemotherapy, which can pave the way for future studies in HGSOC drug resistance practices.

## 1 Introduction

High-Grade Serous Ovarian Carcinoma (HGSOC) is the most prevalent and lethal subtype of ovarian cancer. According to data from the World Health Organization, approximately 225,000 new ovarian cancer cases are reported globally each year, with HGSOC accounting for the vast majority, and its mortality rate ranking among the highest of all gynecological malignancies (1). In the United States, data from the Surveillance, Epidemiology, and End Results (SEER) database of the National Cancer Institute indicate that the five-year survival rate for ovarian cancer patients is merely 49.1% (2).

HGSOC is typically diagnosed at an advanced stage, a phenomenon that results in an extremely poor prognosis, as the absence of early diagnosis and effective treatment contributes to a continuous increase in mortality rates (3). The incidence of HGSOC varies significantly across diverse regions and populations. Epidemiological studies indicate that HGSOC constitutes 70% to 80% of all ovarian cancer cases (4). Despite increased research efforts on ovarian cancer in recent years, the five-year survival rate for HGSOC has demonstrated almost no significant improvement over the past thirty years, reflecting the complexity and challenges associated with its treatment (5).Globally, the impact of HGSOC is particularly severe, especially in developing countries, where many patients are diagnosed at advanced stages due to the absence of effective screening and early diagnostic methods (4). In certain regions, the mortality rate of HGSOC can reach as high as 70% (6). Consequently, establishing effective screening and early diagnostic strategies is crucial for reducing mortality rates associated with HGSOC.

The clinical manifestations of HGSOC are typically not apparent in the early stages, with patients potentially experiencing non-specific symptoms such as abdominal distension, abdominal pain, decreased appetite, and weight changes, which render early diagnosis particularly challenging. Patients with advanced HGSOC frequently present with severe symptoms, including ascites, gastrointestinal symptoms, and systemic weakness, which significantly impact the quality of life of patients (7). Factors contributing to the late-stage diagnosis of HGSOC include the absence of effective screening methods, the subtlety of early symptoms, and patients' tendency to overlook these symptoms. Therefore, early diagnosis is essential for enhancing patient prognosis.

The standard treatment protocol for HGSOC typically involves combination chemotherapy utilizing platinum-based drugs (such as cisplatin or carboplatin) in conjunction with paclitaxel. While this chemotherapy regimen demonstrates favorable response rates in most patients, the emergence of drug resistance remains a primary challenge (8). Initial treatment response rates are typically high; however, approximately 70% of patients experience recurrence, indicating chemotherapy resistance (8). The mechanisms of drug resistance in HGSOC are complex, involving multiple factors, including alterations in the tumor microenvironment, genetic mutations, and drug efflux mechanisms. The emergence of drug resistance significantly reduces the survival rates of HGSOC patients, particularly following recurrence, where patient prognosis is generally poor. The majority of HGSOC patients experience recurrence following initial treatment, with a recurrence rate as high as 70% (9). This elevated recurrence rate complicates the treatment of HGSOC. Future research should concentrate on elucidating the mechanisms underlying HGSOC recurrence and identifying novel therapeutic strategies to enhance patient prognosis and survival rates. Comprehending the mechanisms of drug resistance is essential for enhancing treatment outcomes. Through comprehensive research into resistance mechanisms, researchers can establish a theoretical foundation for the development of novel therapies, ultimately improving patient prognosis. By investigating drug resistance, new biomarkers and therapeutic targets can be identified, thereby providing a basis for personalized treatment and ultimately enhancing patient outcomes. Through interdisciplinary collaboration that integrates knowledge and technologies from various disciplines, research progress in HGSOC can be advanced, yielding new insights and directions for future treatment strategies.

In this review, we firstly described the cellular and molecular mechanisms involved in drug resistance in HGSOC, and summarized the novel diagnostic and therapeutic strategies for detection and treatment of drug resistance in HGSOC, shedding light on the future pre-clinical and clinical investigations targeting HGSOC.

## 2 Biological basis of drug resistance in HGSOC

The molecular subtypes of High-Grade Serous Ovarian Carcinoma (HGSOC) primarily include proliferative, immunoreactive, stromal, and differentiated subtypes, which exhibit significant differences in gene expression, prognosis, and treatment response. For instance, the stromal subtype is generally associated with a poorer prognosis, whereas the immunoreactive subtype exhibits improved survival rates (10, 11).

Through integrated multi-omics data analysis, researchers have discovered that the molecular characteristics of HGSOC differ not only at the genomic level but also display unique alterations at the transcriptomic and epigenetic levels. These differences in molecular features reflect the distinct biological behaviors of tumor cells and their varied responses to treatment, suggesting the clinical necessity of developing personalized treatment strategies based on molecular subtypes.

Different molecular subtypes of HGSOC also exhibit significant variations in clinical manifestations. Clinical studies have shown that patients with the stromal subtype generally present with more advanced disease at diagnosis and have a poorer prognosis, whereas patients with the immunoreactive subtype may be diagnosed at earlier stages and tend to respond more favorably to standard treatments (12).

Furthermore, the tumor microenvironment (TME) of HGSOC displays distinct characteristics across various subtypes. For instance, the degree of immune cell infiltration and the activity of cancer-associated fibroblasts (CAFs) demonstrate significant variations among subtypes, which may potentially impact treatment efficacy and patient prognosis (13).

Therefore, understanding the clinical manifestations and biological characteristics of various subtypes is essential for developing effective treatment strategies. By implementing molecular subtyping in clinical practice, more precise prognostic assessments and personalized treatment plans can be provided for patients, thereby improving the overall survival rates and quality of life for HGSOC patients.

### 2.1 p53 mutation

The p53 gene is one of the most frequently mutated genes in human cancers, with a mutation rate exceeding 50%. In various tumor types, p53 mutations not only result in the loss of its tumor suppressor function but may also confer new oncogenic functions to the mutated p53, a phenomenon termed “gain-of-function” (GOF) (14). In HGSOC, the p53 mutation rate exceeds 96%, underscoring its significance in this cancer type (15, 16). The elevated mutation rate in HGSOC reflects the complexity of its biological characteristics and tumor microenvironment. These mutations not only impact tumor cell survival and proliferation but may also influence the tumor's metastatic potential and treatment response. Consequently, p53 mutations are regarded as important biomarkers for HGSOC, potentially providing new diagnostic and therapeutic targets for clinical practice (17).

The p53 protein plays a crucial role in cell cycle regulation and apoptosis. The normal p53 protein can inhibit tumor development by inducing cell cycle arrest and promoting apoptosis. The mutated p53 protein often loses its ability to regulate the G1/S and G2/M checkpoints, not only permitting tumor cells to continue proliferating under DNA damage or other stress conditions but also potentially promoting tumor growth and metastasis by activating other oncogenic pathways and altering the tumor microenvironment to enhance invasiveness and metastatic potential (18, 19). The presence of p53 mutations in HGSOC is associated with poor patient prognosis, and the type of mutation and expression pattern may influence the tumor's biological behavior and chemotherapy resistance (18). Furthermore, the mutated p53 protein may further influence the apoptosis process through interactions with other signaling pathways, increasing tumor cell resistance to chemotherapy and radiotherapy (20, 21). Comprehensive research into the mechanisms of p53 mutations in HGSOC is of great significance for developing new therapeutic strategies. Therefore, treatment approaches targeting p53 mutations, such as restoring its normal function or employing synthetic lethality strategies, may offer new directions for HGSOC treatment.

### 2.2 Tumor microenvironment characteristics

The mechanisms of drug resistance in HGSOC are complex and diverse, and recent studies have demonstrated a close association between its molecular characteristics and drug resistance. The application of single-cell RNA sequencing (scRNA-seq) technology has revealed a specific cisplatin-resistant cell subpopulation (E0) in HGSOC, which is linked to poor prognosis. E0 cells display malignant characteristics related to drug resistance and promote tumor cell growth through interactions with fibroblasts and endothelial cells, while simultaneously suppressing immune cell infiltration (22).

Furthermore, EZH2, a key component of the Polycomb Repressive Complex 2 (PRC2), is closely linked to drug resistance in HGSOC. Research has shown that in HGSOC with a deficiency of tumor-infiltrating lymphocytes, EZH2 expression in cisplatin-resistant tumors is nearly twice that of normal tumors (23). The tumor microenvironment (TME) in HGSOC also plays a critical role in drug resistance. Alterations in cancer-associated fibroblasts and immune cells within the tumor microenvironment can significantly influence HGSOC progression and treatment response. Furthermore, intercellular interactions and activated signaling pathways within the tumor microenvironment influence the chemotherapeutic response and drug resistance. Research indicates that the HGSOC tumor microenvironment displays significant molecular alterations that are closely linked to tumor cell drug resistance (24).

The high intratumoral heterogeneity (ITH) in HGSOC results in diverse molecular characteristics at different stages, consequently affecting its sensitivity and resistance to chemotherapy (25). The relationship between molecular characteristics and drug resistance in HGSOC constitutes a complex network involving multiple molecular pathways and cellular interactions. Therefore, further exploration of how these molecular features influence treatment responses in HGSOC may yield more precise clinical treatment strategies.

The composition and structure of the tumor microenvironment directly influence drug delivery efficiency. The abnormal extracellular matrix and tumor vasculature surrounding tumor cells result in uneven drug distribution within the tumor, thereby reducing the effective drug concentration (26, 27). The TME of HGSOC comprises multiple cell types and matrix components that interact and influence tumor growth and drug resistance. The primary components include tumor cells, cancer-associated fibroblasts (CAFs), immune cells such as macrophages, T cells, and B cells, vascular endothelial cells, and extracellular matrix (ECM) components.

CAFs play a critical role in tumor progression and drug resistance by secreting growth factors and cytokines that promote tumor cell proliferation and migration. Simultaneously, they significantly influence immune cell function, thereby creating an immunosuppressive environment that further enhances drug resistance (28). The distribution of immune cells within the tumor microenvironment is equally significant. Immunosuppressive cells, such as regulatory T cells and M2 macrophages, can suppress anti-tumor immune responses, thereby facilitating tumor growth and drug resistance (29, 30). These cells actively contribute to creating a protective ecosystem that shields tumor cells from therapeutic interventions (31).

Furthermore, immune cells in the tumor microenvironment can potentially influence drug metabolism and elimination by releasing specific cytokines and chemical substances, thereby exacerbating drug resistance mechanisms (32–34). The complex interactions among CAFs, immune cells, and tumor cells create a dynamic microenvironment that continuously challenges therapeutic effectiveness, illustrating the intricate cellular and molecular networks that contribute to treatment resistance in high-grade serous ovarian cancer (35).

Cancer stem cells (CSCs) are a population of tumor cells with self-renewal and differentiation potential; in HGSOC, they are regarded as one of the primary causes of drug resistance and recurrence. These cells can survive chemotherapy and regenerate tumors, resulting in treatment failure (36). Furthermore, HGSOC tumor stem cells can evade chemotherapeutic drugs through multiple mechanisms, such as enhancing DNA repair capabilities and upregulating the expression of drug efflux pumps (22).

Tumor stem cells in HGSOC typically express specific markers such as CD133, ALDH, and OCT4, which are closely linked to tumor invasiveness and drug resistance. The presence of tumor stem cells is directly correlated with chemotherapy resistance in HGSOC, and these cells can evade drug effects through multiple mechanisms (37–39). The relationship between HGSOC tumor stem cell markers and drug resistance is primarily reflected in their self-renewal capacity and resistance to chemotherapeutic drugs. For instance, high expression of ALDH1 is correlated with tumor stem cell characteristics and closely linked to drug resistance. The expression levels of these markers may serve as potential biomarkers for predicting drug resistance in HGSOC patients (28).

**Figure 1:**
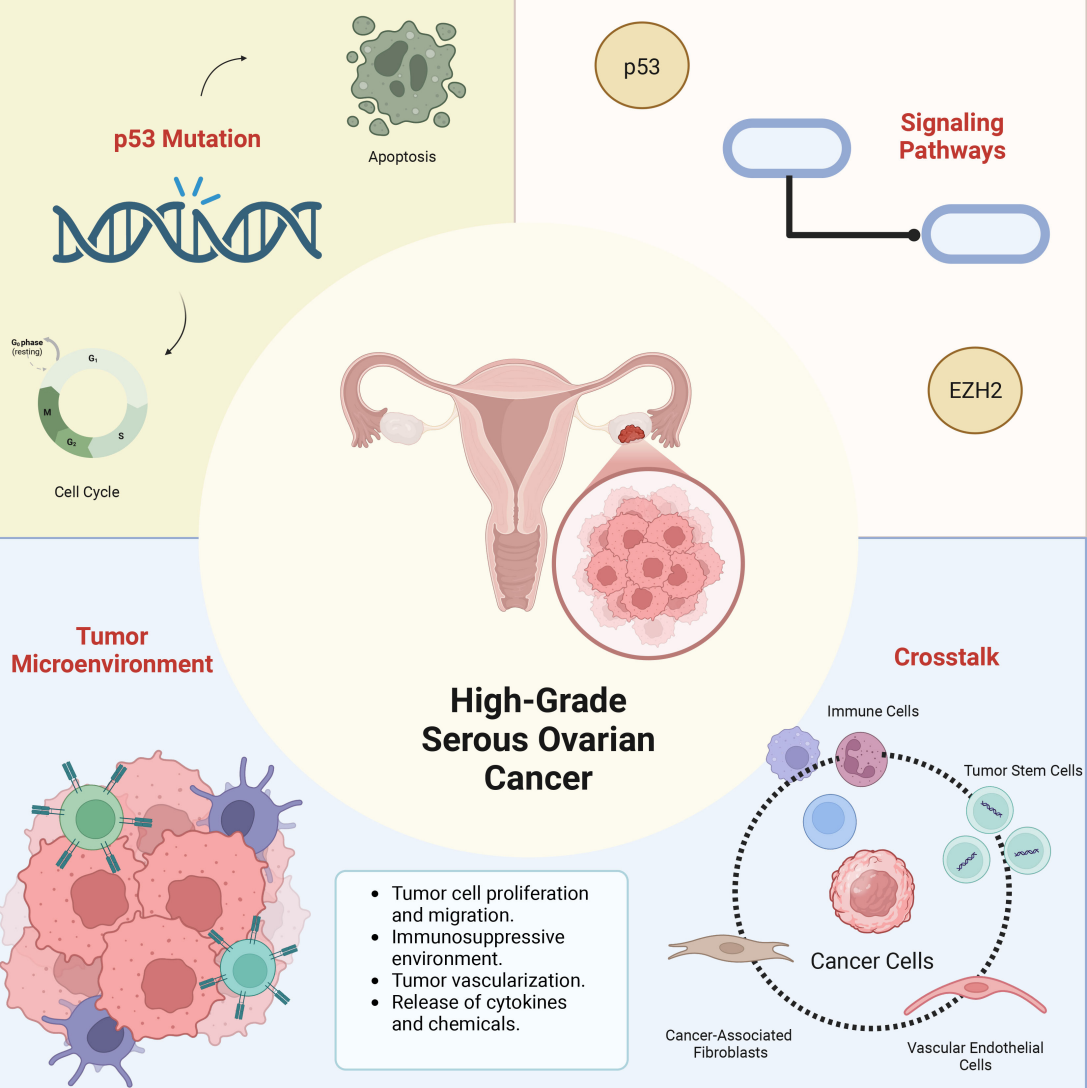
The main biological basis for inducing resistance to HGSOC. In this image, we describe the roles of key gene mutations, critical signaling pathways, and tumor microenvironment interactions as the main biological mechanisms involved in the resistance of HGSOC. We have provided a detailed description of the content included in the image in the corresponding text. HGSOC, High-Grade Serous Ovarian Cancer; p53, Tumor Protein p53; EZH2, Enhancer of Zeste Homolog 2.

In our comprehensive exploration of HGSOC drug resistance mechanisms, we identified a complex and diverse biological foundation involving abnormalities in multiple cellular signaling pathways and alterations in the tumor microenvironment. These factors not only influence tumor cell responses to chemotherapeutic drugs but also play a critical role in patient clinical prognosis. Drug resistance is not attributed to a single factor; rather, it results from the interaction of multiple biological mechanisms. We have illustrated the contributing factors of HGSOC drug resistance in **Figure 1** and presented the various molecular subtypes and clinical prognostic characteristics of HGSOC in the TCGA database in **Table 1** (40).

**Table 1:** Transcriptional subtypes and clinical characteristics of HGSOC.

Subtypes	Characteristics	Immune Infiltration Level	Chemotherapy Sensitivity	Risk (5-year survival %)
Differentiated	High genomic integrity, low ploidy and subclonality	High	Generally sensitive	High (34%)
Immunoreactive	Strong immune cell infiltration	High	Potentially sensitive, varies by patient	Low (50%)
Mesenchymal	Associated with changes in tumor microenvironment	Moderate to high	Lower sensitivity	Very High (20%)
Proliferative	Extreme genomic instability	Low	Generally less responsive	High (34%)

## 3 Major mechanisms of drug resistance in HGSOC

Drug resistance is not merely a biological phenomenon; rather, it presents a critical challenge in clinical treatment. The mechanisms of drug resistance in HGSOC encompass multiple pathways, including drug efflux, cell membrane transport, DNA damage repair, the role of non-coding RNAs, and the impact of the tumor microenvironment on immune escape. This diversity renders the phenomenon of drug resistance exceedingly complex, necessitating the adoption of multidimensional analytical and strategic approaches in both research and clinical applications.

### 3.1 Drug efflux and cell membrane transport

Cell membrane transporters play a pivotal role in drug uptake. By mediating the entry of drug molecules, these transporters significantly influence drug bioavailability and therapeutic efficacy. Different types of transporters exhibit varying affinities and specificities for drugs, resulting in differences in the absorption efficiency of identical drugs across various cell types. For instance, certain anticancer drugs enter HGSOC cells via specific transporters, and the expression levels of these transporters may vary due to tumor-induced drug resistance (41).Drug-resistant cells typically exhibit the overexpression of transporters, leading to increased drug efflux and reduced intracellular drug concentrations (42). Furthermore, functional changes in transporters may be associated with alterations in the tumor microenvironment, thereby affecting tumor cell survival and proliferation. By regulating transporter activity, drug accumulation in tumor cells can be enhanced, thereby improving therapeutic efficacy (43, 44).

Alterations in cell membrane transporter expression significantly impact drug uptake. ATP-binding cassette (ABC) transporters actively expel drugs by utilizing ATP, thereby reducing intracellular drug concentrations and contributing to the development of drug resistance (45). In HGSOC, drug-resistant cells frequently exhibit the overexpression of transporters, resulting in increased drug efflux and decreased intracellular drug concentrations, thereby diminishing drug efficacy. This indicates that the upregulation of ABC transporters is closely associated with chemotherapy resistance in HGSOC. For example, ABCB1 transporters are frequently upregulated in HGSOC cells, resulting in increased resistance to multiple chemotherapeutic agents. High expression of ABCA1 is regarded as a marker of poor prognosis in HGSOC patients, suggesting its significant role in the development of drug resistance (45). Simultaneously, the expression of ABCB1 and ABCC1 significantly increases in drug-resistant HGSOC cells, and the elevated expression of these transporters is closely associated with shortened patient survival and poor chemotherapy response (46). Additionally, the transcriptional fusion forms of ABCB1 transporters are closely associated with drug resistance in HGSOC cells. Alterations in the expression of these transporters not only affect drug uptake but may also lead to metabolic changes, further promoting tumor progression (47).

Therefore, understanding the mechanisms of action of transporters is crucial for optimizing drug design and enhancing therapeutic efficacy. Intervention strategies targeting transporters may represent a novel approach to overcoming HGSOC drug resistance. In summary, targeted therapy against these transport proteins may yield new insights into overcoming HGSOC drug resistance, and further research into their expression mechanisms and functions will facilitate the development of more effective treatment strategies.

### 3.2 DNA damage repair

Research on drug resistance mechanisms in HGSOC is gradually revealing the phenomenon of tumor cell reprogramming in DNA repair pathways. HGSOC cells counteract chemotherapy-induced DNA damage by activating alternative DNA repair mechanisms (1, 48). Homologous Recombination Deficiency (HRD) plays a critical role in HGSOC drug resistance, rendering tumor cells more sensitive to DNA-damaging agents such as platinum compounds and PARP inhibitors. This heightened sensitivity primarily arises from the cells' lack of effective DNA repair mechanisms. The BRCA1 and BRCA2 genes are particularly crucial for DNA damage repair in HGSOC, especially in the Homologous Recombination Repair (HRR) mechanism. The loss of BRCA1 and BRCA2 leads to the impairment of homologous recombination repair functions, rendering tumor cells unable to effectively repair DNA damage, consequently causing genomic instability and tumor progression (32).

When these cells experience DNA damage, they cannot repair it through the HRR pathway, leading to cell death (49). Research demonstrates that HRD cells exhibit significantly increased cell death rates when treated with platinum chemotherapy or PARP inhibitors, indicating that HRD is a significant predictor of treatment response (50). Studies reveal that tumor cells with BRCA1/2 deficiencies exhibit high sensitivity to PARP inhibitors, a phenomenon termed “synthetic lethality,” meaning that inhibiting PARP in a BRCA-deficient background results in tumor cell death (51). Consequently, BRCA gene mutations are not only critical pathogenic factors in HGSOC but also therapeutic targets.

However, as treatment progresses, tumor cells may acquire drug resistance through various mechanisms. For instance, research shows that HRD tumor cells, after platinum treatment, may evade drug effects by restoring HRR functionality or upregulating multi-drug resistance-related genes (52, 53). Despite HRD rendering HGSOC cells sensitive to certain treatments, resistance mechanisms gradually emerge during therapy. Studies have found that HRD cells may develop drug resistance by recovering homologous recombination repair functionality, typically involving reverse mutations in BRCA1/2 genes or mutations in other DNA repair genes (54).

Furthermore, changes in the tumor microenvironment, intercellular signaling, and gene expression alterations can also promote the development of resistance. Reverse mutations in BRCA genes represent a critical mechanism of PARP inhibitor resistance. As treatment progresses, tumor cells in some HGSOC patients may restore BRCA functionality through mutations, thereby regaining homologous recombination repair capabilities. These reverse mutations enable tumor cells to resist PARP inhibitors, resulting in treatment failure (55). Research indicates that BRCA1/2 gene reverse mutations not only affect the DNA repair abilities of tumor cells but may also impact their sensitivity to other treatments (56). Additionally, some studies suggest that tumor cells enhance PARP inhibitor resistance by upregulating the activity of the PI3K/Akt pathway (57). Tumor cells develop PARP inhibitor resistance through various mechanisms, including gene mutations, epigenetic modifications, and alterations in signaling pathways. For instance, research has found that tumor cells may reduce intracellular PARP inhibitor concentrations by upregulating multi-drug resistance-related genes or evade drug effects by altering cell cycle regulatory mechanisms (58).

In HGSOC, the activation of alternative repair mechanisms is a critical component of drug resistance. Research reveals that platinum-resistant HGSOC cells often exhibit suppression of Homologous Recombination (HR) repair pathways, while alternative repair mechanisms, such as Non-Homologous End Joining (NHEJ), are activated (59). This enhancement of alternative repair mechanisms enables tumor cells to effectively repair DNA damage, thereby evading the cytotoxic effects of chemotherapeutic agents. For instance, overexpression of EHMT1 and EHMT2 is associated with PARP inhibitor resistance, as they influence gene expression by modulating the methylation status of histone H3, thereby promoting tumor cell survival and proliferation (60).

Single-cell RNA sequencing has revealed specific drug-resistant cell subpopulations in HGSOC that promote their growth and drug resistance through interactions with fibroblasts and endothelial cells in the tumor microenvironment (22). These findings provide new insights into understanding HGSOC resistance mechanisms and point toward future therapeutic strategies. The specific molecular characteristics of HRD tumor cells, such as TP53 mutations and genomic instability, further enhance their sensitivity to DNA-damaging agents. Consequently, HRD is not only a critical feature of tumor biology but also a key consideration in clinical treatment strategies.

### 3.3 Apoptosis and autophagy

In the apoptotic pathways of tumor cells and HGSOC cells, key proteins such as the Bcl-2 family, p53, and caspases play crucial roles (61). The interactions among these proteins form a complex network that regulates tumor cell survival and apoptosis. Bcl-2 family proteins regulate apoptosis by modulating mitochondrial outer membrane permeability, with the relative expression levels of Bcl-2 and Bax determining cell survival or death (62). In tumor cells and HGSOC cells, overexpression of the anti-apoptotic protein Bcl-2 can inhibit apoptotic signals, thereby enhancing tumor cell resistance to chemotherapeutic agents (63).

p53, as a tumor suppressor, promotes apoptosis by inducing the expression of apoptosis-related genes, particularly in cases of DNA damage (64). However, in tumor cells, mutations or inactivation of p53 prevent effective responses to DNA damage, leading to reduced sensitivity to chemotherapeutic agents (65). Caspases, as executioners of apoptosis, participate in signal transduction during apoptosis, activating a series of downstream effector molecules that ultimately lead to cell death (66). In tumor cells and HGSOC cells, reduced caspase activity can impair the execution of apoptosis, thereby rendering tumor cells resistant to treatment (67). Functional abnormalities of these key proteins often lead to the development of drug resistance, causing HGSOC patients to frequently experience poor treatment outcomes with standard therapies.

Autophagy plays a dual role in the drug resistance of tumor cells and HGSOC. On one hand, autophagy can maintain cellular metabolic balance and survival by clearing damaged organelles and proteins, thereby promoting tumor cell survival and drug resistance in certain contexts (68). Conversely, autophagy can also inhibit tumor cell growth by promoting apoptosis (69). Therefore, the activity and regulatory state of autophagy significantly impact tumor cell drug resistance.

The interaction between autophagy and apoptosis is particularly complex in tumor cells and HGSOC cells. Studies have shown that autophagy can influence survival by regulating the expression of apoptosis-related proteins (70). For instance, autophagy activation may lead to Bcl-2 degradation, thereby promoting apoptosis (71). Furthermore, autophagy can enhance apoptotic signal transmission by clearing apoptosis signal inhibitors, thus facilitating tumor cell death (72).

The interaction between autophagy and apoptosis significantly impacts drug efficacy. Research indicates that autophagy activation may enhance drug resistance in tumor cells during chemotherapy, as autophagy can clear drug-induced cellular damage, thereby protecting tumor cell survival (73). Conversely, inhibiting autophagy can enhance the effects of chemotherapeutic agents and promote tumor cell apoptosis. Therefore, combining autophagy inhibitors with chemotherapeutic agents may improve treatment outcomes for HGSOC. Understanding this interaction provides a critical theoretical basis for developing new therapeutic strategies.

### 3.4 Epigenetic modifications

In recent years, research has demonstrated that epigenetic modifications play a crucial role in the drug resistance of HGSOC, including changes in DNA methylation, histone modifications, and non-coding RNA expression. Research has revealed that abnormal regulation of N6-methyladenosine (m6A) modification in HGSOC cells is closely associated with tumor occurrence and metastasis (74, 75). Furthermore, overexpression of histone lysine N-methyltransferases EHMT1 and EHMT2 is linked to PARP inhibitor resistance. These enzymes influence gene expression by regulating the methylation status of histone H3, thereby promoting tumor cell survival and proliferation (60). Additionally, KRAS gene amplification may be one reason for the resistance of BRCA2-deficient HGSOC to PARP inhibitors. The combined use of PLK1 inhibitors can restore sensitivity to PARP inhibitors, suggesting that epigenetic modifications may regulate drug resistance by affecting signaling pathways (32). Therefore, understanding these mechanisms of epigenetic modification will provide potential targets for developing new therapeutic strategies.

Furthermore, the activation of the mTOR signaling pathway is associated with the upregulation of FANCD2, which may regulate sensitivity to platinum-based drugs by affecting the expression of DNA crosslink repair proteins, thereby influencing drug resistance in HGSOC (76). These research findings demonstrate that epigenetic modifications play a crucial role not only in tumor occurrence and development but also in therapeutic resistance, providing new insights for future treatment strategies.

### 3.5 Non-coding RNAs

In the complex pathogenesis of High-Grade Serous Ovarian Carcinoma (HGSOC), multiple non-coding RNAs (ncRNAs) play crucial regulatory roles (77, 78). Non-coding RNAs primarily include microRNAs (miRNAs), long non-coding RNAs (lncRNAs), and circular RNAs (circRNAs). These non-coding RNAs perform important biological functions within cells, including regulating gene expression and participating in cell proliferation, differentiation, and apoptosis (79). Research indicates that the expression levels of non-coding RNAs in HGSOC are closely associated with tumor occurrence, development, and response to chemotherapy (77, 80, 81). In HGSOC, non-coding RNAs such as miRNAs, lncRNAs, and circRNAs regulate tumor occurrence and development through various mechanisms.

MicroRNAs (miRNAs) play a crucial role in post-transcriptional regulation by binding to target gene mRNAs, inhibiting their translation or promoting their degradation (80, 81). miRNAs directly influence drug resistance in High-Grade Serous Ovarian Carcinoma (HGSOC) by regulating genes associated with drug metabolism, apoptosis, and DNA repair. The upregulation of certain miRNAs is associated with platinum drug resistance in HGSOC cells, which may be related to the expression levels of their target genes (82). miRNAs such as miR-16, miR-17, and miR-93 have been found to be closely related to chemotherapeutic responses in HGSOC and can predict patient sensitivity to chemotherapy (83, 84).

Some lncRNAs promote tumor cell proliferation and metastasis by regulating gene transcription and translation (85). Furthermore, lncRNAs have been found to be closely associated with HGSOC drug resistance, with certain lncRNAs promoting tumor cell survival and drug resistance by modulating the tumor microenvironment and cell signaling pathways (86). Additionally, lncRNAs and circRNAs participate in HGSOC biological processes through various mechanisms, influencing tumor cell growth and drug resistance (87).

circRNA as a novel non-coding RNA, has gradually revealed its functions in High-Grade Serous Ovarian Carcinoma (HGSOC). Research has discovered that circRNA can regulate target gene expression by competitively binding to miRNAs, thereby influencing tumor cell biological behaviors (87). Simultaneously, circRNA also plays a role in drug resistance through competitive miRNA binding, altering target gene expression and consequently affecting drug resistance (88). Therefore, in-depth research on the role of non-coding RNAs in the mechanisms of HGSOC drug resistance can help develop new therapeutic strategies and improve patient treatment outcomes.

**Figure 2:**
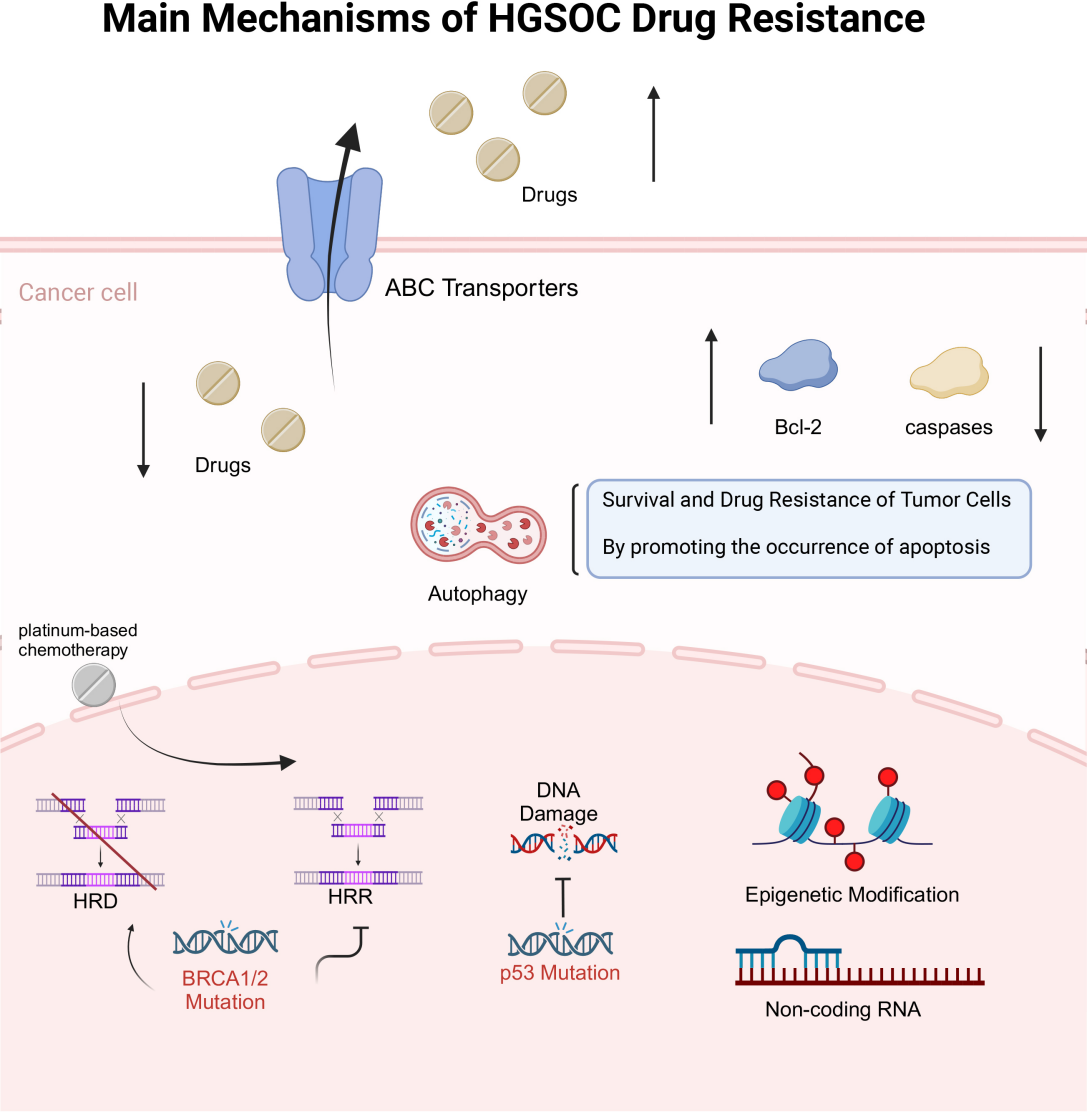
Primary Drug Resistance Mechanisms in HGSOC. In this figure, we describe the mechanisms by which drug transport and efflux pathways, homologous recombination repair pathways, apoptosis and autophagy pathways, epigenetic pathways, and non-coding RNA pathways influence the resistance of HGSOC. We have provided a detailed description of the content included in the image in the corresponding text. HGSOC, High-Grade Serous Ovarian Cancer; p53, Tumor Protein p53; HRD, Homologous Recombination Deficiency; HRR, Homologous Recombination Repair; ABC transporter, ATP-Binding Cassette transporter; Bcl-2, B-cell lymphoma 2.

When summarizing the diversity and complexity of drug resistance mechanisms in High-Grade Serous Ovarian Carcinoma (HGSOC), it is evident that drug resistance is not merely a biological phenomenon but a critical challenge in clinical treatment. The drug resistance mechanisms of HGSOC involve various pathways, including gene mutations, epigenetic alterations, regulation of cell signaling pathways, and influences from the tumor microenvironment. This diversity renders drug resistance extremely complex, necessitating multi-dimensional analytical and strategic approaches in both research and clinical applications. The primary drug resistance mechanisms of HGSOC are illustrated in **Figure 2**.

## 4 Detection and diagnostic strategies for HGSOC drug resistance

HGSOC is a highly aggressive and drug-resistant tumor, making research into its drug resistance mechanisms crucial for improving patient prognosis. In recent years, as the understanding of HGSOC drug resistance mechanisms has deepened, related molecular markers and companion diagnostic strategies have gradually become important components of clinical management.

Homologous recombination deficiency (HRD) caused by BRCA1/2 gene mutations leads to resistance to platinum-based chemotherapeutic agents, a phenomenon particularly evident in HGSOC (54). The presence of BRCA1/2 mutations is closely associated with patient responses to platinum-based chemotherapy, with mutated patients typically showing good initial treatment responses that gradually develop resistance over time (89). Therefore, detecting BRCA1/2 mutations can help predict patient chemotherapy responses and guide subsequent treatment strategies, such as the use of PARP inhibitors and other targeted therapies. BRCA1/2 detection has become an important component of personalized treatment for HGSOC patients, contributing to optimized clinical decision-making and improved patient survival rates.

The Homologous Recombination Deficiency (HRD) Score is a tool for predicting cancer patients' chemotherapy responses by assessing genomic instability (90). The HRD score reflects cancer cells' deficiencies in DNA repair, with particularly significant correlations to platinum-based chemotherapy resistance in HGSOC. Research indicates that patients with high HRD scores typically demonstrate better responses to platinum-based chemotherapy and longer survival periods (91). Furthermore, the HRD score can be used to screen patients suitable for PARP inhibitor treatment (92). By evaluating the HRD score, clinicians can develop more personalized treatment plans, thereby improving therapeutic outcomes. The clinical application of HRD scoring has gradually become an important method for detecting drug resistance in HGSOC, providing patients with more treatment options (93).

Tumor Mutation Burden (TMB), defined as the number of mutations per million base pairs in tumor cells, has been recognized in recent years as an important biomarker for predicting responses to immunotherapy (94). In HGSOC, TMB is closely associated with patient prognosis and responses to immune checkpoint inhibitors. Research has found that patients with high TMB typically exhibit better responses to immunotherapy and longer survival periods (95). TMB detection can help identify patients who may benefit from immunotherapy, especially in cases of resistance to platinum-based chemotherapy (96). Furthermore, combining TMB with HRD scoring can provide clinicians with more comprehensive patient information, thereby optimizing treatment plans and improving patient survival rates and quality of life (97).

With the in-depth investigation of drug resistance mechanisms in HGSOC, the discovery of emerging biomarkers provides novel insights into managing resistance. For instance, miRNAs associated with chemotherapy resistance, such as miR-23a-3p and miR-181c-5p, have been found to influence patient prognosis (82). The potential application of these emerging markers lies in their ability to serve as early predictors of drug resistance, enabling clinicians to adjust treatment strategies in a timely manner and improve patient survival rates and quality of life. Furthermore, specific gene mutations and epigenetic alterations may be associated with HGSOC drug resistance, offering new therapeutic targets for future treatment strategies (98). In the research on drug resistance mechanisms of HGSOC, studies focusing on specific drug-resistant proteins and exosomal markers have gradually gained prominence. For instance, amplification of the CCNE1 gene is closely associated with resistance to PARP inhibitors and platinum-based agents (99). Furthermore, miRNAs and proteins within exosomes are considered critical biomarkers of drug resistance, capable of reflecting the drug-resistant state and microenvironmental changes in tumors (84). These marker studies provide novel insights into HGSOC drug resistance and offer potential clinical biomarkers for monitoring resistance and guiding therapeutic strategies. The clinical prospects for novel drug resistance biomarkers in HGSOC are promising, and with a deeper understanding of resistance mechanisms, individualized treatment strategies based on these markers can be developed in the future. A drug resistance prediction model based on miRNA expression profiles may become a crucial tool in clinical practice, helping physicians select the most appropriate treatment plan (100). Furthermore, by integrating emerging genomics and transcriptomics technologies, a more comprehensive assessment of patient drug resistance status can be achieved, thereby realizing the goal of precision medicine (101).

Traditional tissue biopsy remains a critical tool in HGSOC drug resistance research (102). Its primary advantage lies in providing direct tumor tissue samples, allowing pathologists to conduct detailed morphological and molecular biological analyses, thereby establishing a basis for individualized treatment plans (103). However, traditional tissue biopsy also presents significant drawbacks, including high invasiveness, patient discomfort, and insufficient sample representation (104). Moreover, due to tumor heterogeneity, a biopsy from a single site may not represent the entire tumor's biological characteristics, potentially leading to misinterpretation of treatment responses, especially when tumor cells metastasize or develop drug resistance. Therefore, biopsy results must be interpreted cautiously in clinical applications.

With the development of liquid biopsy technology, the indications for tissue biopsy are gradually being reconsidered. For patients with multiple metastases or significant tumor heterogeneity, liquid biopsy may provide more comprehensive biological information (105). Thus, when selecting a biopsy method, it is necessary to comprehensively consider the patient's specific conditions and tumor characteristics to ensure accurate diagnosis and effective treatment strategies. Liquid biopsy, as an emerging detection technique, offers the advantages of non-invasiveness and real-time monitoring (105). By analyzing circulating tumor cells (CTCs), circulating tumor DNA (ctDNA), and exosomes in blood or other bodily fluids, liquid biopsy can provide real-time information about dynamic changes in tumors (106). This method not only reduces patient suffering but also enables timely reflection of tumor responses to treatment, helping physicians adjust treatment plans and improve therapeutic outcomes. The application of liquid biopsy in monitoring HGSOC drug resistance is increasingly widespread. Studies have shown that detecting specific mutations in ctDNA can effectively predict patient resistance to chemotherapy and targeted therapy (107, 108). Additionally, liquid biopsy can monitor changes in tumor burden, providing clinicians with timely feedback to optimize treatment strategies. Compared to traditional tissue biopsy, liquid biopsy demonstrates higher sensitivity and specificity in monitoring drug resistance, better accommodating tumor heterogeneity.

Multi-omics detection methods combine technologies such as genomics, transcriptomics, and proteomics, offering new perspectives for investigating the mechanisms of HGSOC drug resistance (109, 110). These methods can comprehensively analyze tumor molecular characteristics, revealing interactions between different omics levels and providing a basis for individualized treatment. By integrating diverse omics data, researchers can identify drug resistance-related biomarkers, subsequently optimizing treatment plans and improving patient survival rates. In multi-omics detection, the combination of next-generation sequencing (NGS), polymerase chain reaction (PCR), and immunohistochemistry (IHC) provides powerful tools for investigating the mechanisms of HGSOC drug resistance. NGS enables high-throughput detection of genomic mutations in tumors, PCR is used to verify specific gene expressions, and IHC can assess protein expression levels (16, 111). The integration of these technologies enhances detection sensitivity and accuracy while providing more comprehensive biological information about tumors, promoting the development of individualized treatment.

In conclusion, the mechanisms of HGSOC drug resistance are complex and diverse, involving the application of multiple molecular biomarkers and detection technologies. By comprehensively evaluating biomarkers such as BRCA1/2 mutations, HRD scores, TMB, and liquid biopsy, clinicians can develop better individualized treatment plans, improving patient survival rates and quality of life. Future research should continue exploring emerging biomarkers and their potential applications in monitoring drug resistance to further improve treatment outcomes for HGSOC patients. The primary HGSOC drug resistance detection biomarkers and their related clinical information are summarized in **Table 2**.

**Table 2:** Key biomarkers in HGSOC and information on resistance and prognosis.

Biomarker	Characteristics	Mechanism of Resistance	Prognosis	Refs.
BRCA1/2	gene mutated	HR repair defects, increasing sensitivity to platinum-based chemotherapy	Favorable but may develop resistance.	(112)
NF1	gene deletion	Acting in conjunction with the inactivation of other tumor suppressor genes	Unfavorable	(89, 113)
TP53	gene mutated	Cell cycle dysregulation, affecting apoptosis	Unfavorable	(113)
PTEN	gene deletion	Activation of the PI3K signaling pathway	Unfavorable	(89)
RB1	gene inactivation	Cell cycle dysregulation	Unfavorable	(89, 113)
MYC	gene amplification	Promoting cell proliferation and survival	Unfavorable	(114)
PAX8	gene amplification	Influence response to targeted therapy	Favorable	(115)
miRNAs	Abnormal expression	Affecting tumor cell survival	Unfavorable	(8)

HGSOC, High-Grade Serous Ovarian Cancer; HR,Homologous recombination; BRCA1, Breast Cancer 1; BRCA2, Breast Cancer 2; NF1, Neurofibromatosis Type 1; TP53, Tumor Protein p53; PTEN, Phosphatase and Tensin Homolog; RB1, Retinoblastoma 1; MYC, Myelocytomatosis Viral Oncogene Homolog; PAX8, Paired Box Gene 8; miRNAs, MicroRNAs.

## 5 Therapeutic advances in overcoming resistance in HGSOC

In recent years, treatment strategies for overcoming HGSOC drug resistance have continuously advanced, encompassing multiple aspects, including combination chemotherapy, targeted therapy, personalized treatment, immunotherapy, and emerging epigenetic and oncolytic virus therapies.

The standard treatment protocol for HGSOC typically involves the combined use of platinum drugs (such as cisplatin or carboplatin) with paclitaxel (116). This combination therapy has been widely applied clinically, aiming to improve patients' progression-free survival (PFS) and overall survival (OS). Research indicates that platinum drugs interfere with DNA synthesis and repair mechanisms, leading to cancer cell death, while paclitaxel stabilizes microtubules, preventing cell division and thereby enhancing chemotherapeutic effects (117, 118). However, approximately 20% of HGSOC patients exhibit intrinsic resistance to this standard chemotherapy regimen, resulting in poor treatment outcomes and compromised prognosis (80, 119).

Chemotherapy effectiveness is influenced by multiple factors, including drug dosage, administration protocols, and timing. Dose-intensive chemotherapy typically increases tumor response rates but is accompanied by higher risks of toxicity. Protocols using higher doses of platinum drugs combined with paclitaxel can partially overcome tumor resistance to drugs and improve patient survival prognosis (120). However, balancing treatment efficacy and toxicity risks requires further investigation. The interval between drug administrations also significantly impacts chemotherapy effectiveness; frequent administration within short periods may decrease patient tolerance, while prolonged intervals might provide tumor cells with opportunities for regrowth (121). Therefore, designing individualized administration protocols is crucial for improving HGSOC chemotherapy success rates.

### 5.1 Traditional protocols

Research on chemotherapy drug resistance has led to new breakthroughs in HGSOC treatment. The mechanisms of drug resistance reversal agents primarily involve regulating signaling pathways related to the cell cycle, apoptosis, and autophagy. These studies provide a theoretical foundation for developing new reversal agents. PDZ-binding kinase (PBK) is closely associated with HGSOC chemotherapy resistance, with its overexpression linked to both chemotherapy resistance and poor prognosis. PBK promotes autophagy by activating the ERK/mTOR signaling pathway, thereby enhancing resistance to platinum drugs (122). TTK protein kinase, a critical cell cycle regulatory factor, shows potential in enhancing sensitivity to platinum drugs in HGSOC cells. Inhibition of TTK interferes with cell cycle progression, increases cell apoptosis, and consequently improves sensitivity to chemotherapeutic drugs (123).

Additionally, PARP inhibitors (PARPi) demonstrate significant clinical efficacy in BRCA-mutated patients (124), but emerging resistance limits their long-term effectiveness (125), Patients may develop resistance through multiple mechanisms, such as secondary BRCA gene mutations or restoration of DNA repair pathways (126). Combination therapy using PARP and ATR inhibitors has shown promising results in clinical trials, particularly in platinum-resistant HGSOC patients (127).

Angiogenesis inhibitors work by blocking tumor neovascularization, thereby limiting tumor growth and metastasis. VEGF pathway inhibitors, such as bevacizumab, have demonstrated potential benefits for HGSOC patients, though resistance remains a challenge (128, 129). Signaling pathway inhibitors, such as MEK inhibitors, have been studied to enhance HGSOC chemosensitivity, significantly increasing cytotoxicity against drug-resistant HGSOC cells when combined with platinum drugs (130, 131).

### 5.2 Emerging protocols

By analyzing patients’ tumor molecular characteristics, more precise treatment strategies can be developed. Based on genomic sequencing and transcriptome analysis, researchers can identify biomarkers associated with drug resistance. Patient-derived organoid (PDO) technology can simulate tumor growth environments *in vitro*, providing more reliable experimental foundations for personalized treatment (132).

In recent years, tumor vaccines have gradually attracted attention as a new immunotherapeutic strategy for HGSOC. Research has found that HGSOC tumor cells can activate immune responses by expressing specific antigens (133). Tumor antigen vaccines and dendritic cell vaccines stimulate T-cell activation and proliferation, enhancing the body’s immune surveillance of tumor cells (134, 135). However, the complex tumor microenvironment of HGSOC presents challenges for the clinical application of vaccines, with tumor-associated macrophages and other immunosuppressive cells potentially inhibiting vaccine-induced immune responses (136). Future research needs to explore optimizing vaccine design and effectively overcoming the immune suppression of the tumor microenvironment.

Cell therapy, as an emerging immunotherapeutic strategy, shows promising potential in HGSOC treatment. CAR-T cell therapy uses genetic engineering to transform patients’ T cells into cells capable of specifically identifying tumor cells, thereby enhancing anti-tumor capabilities (137). CAR-T cell therapy has achieved significant success in other cancer types and is gradually being applied to HGSOC research, with preliminary results showing good efficacy in certain patients (138–140). However, the effectiveness of CAR-T cell therapy is influenced by the tumor microenvironment, and overcoming tumor immune escape mechanisms remains a key focus for future research (141).

Emerging epigenetic therapies provide new insights into HGSOC resistance. Epigenetic modifications, such as DNA methylation, histone acetylation, lactylation, and RNA m6A and m5C modifications, are considered crucial in the occurrence of HGSOC and drug resistance (74). For instance, DNA methyltransferases (DNMT) are typically overexpressed in HGSOC cells, and their inhibitors, such as demethylating drugs (like azacitidine and decitabine), have shown potential therapeutic effects in preclinical studies (80, 142). Moreover, histone deacetylase (HDAC) inhibitors demonstrate anti-tumor activity against HGSOC cells, though clinical trials may encounter off-target effects (143). These epigenetic drugs can potentially reverse drug resistance in HGSOC cells by regulating gene expression, thus improving treatment outcomes.

Oncolytic virus therapy, an emerging cancer treatment approach, has garnered widespread attention in HGSOC research. Oncolytic viruses selectively infect and destroy tumor cells, inducing tumor cell death and activating host immune responses to enhance anti-tumor immunity (144). Studies indicate that oncolytic viruses can be combined with immune checkpoint inhibitors to further enhance the immune system’s attack on tumors (145). Additionally, epigenetic regulators might help tumor cells regain sensitivity to oncolytic viruses, achieving better therapeutic effects (146). Therefore, strategies that combine oncolytic viruses with immunotherapy and epigenetic treatments offer new possibilities for HGSOC treatment, warranting further exploration in future clinical research.

In conclusion, the mechanisms of HGSOC drug resistance are complex and diverse, involving multiple molecular markers and treatment strategies. By comprehensively evaluating combination chemotherapy, targeted therapy, personalized treatment, immunotherapy, and emerging therapies, clinicians can develop better individualized treatment plans, improving patient survival rates and quality of life. Future research should continue exploring emerging markers and their potential applications in monitoring drug resistance to further improve treatment outcomes for HGSOC patients. We have summarized existing and emerging treatment protocols in **Table 3** to highlight the characteristics and application scenarios of different treatment strategies.

**Table 3:** Existing and emerging treatment strategies for HGSOC.

Treatment Strategy	Target	Clinical Trial Phase	Main Challenges
Platinum and Paclitaxel Combination Therapy	DNA synthesis and repair mechanisms; microtubule stabilization	Widely used in clinical practice	About 20% of patients have intrinsic resistance, affecting treatment efficacy and prognosis
Dose-Dense Chemotherapy	DNA synthesis and repair mechanisms; microtubule stabilization	Under investigation	Higher response rates may come with increased toxicity risks
PARP Inhibitors	DNA repair mechanisms	Demonstrated significant clinical efficacy	Resistance phenomena limit long-term efficacy
Angiogenesis Inhibitors,e.g., Bevacizumab	VEGF pathway	Demonstrated potential benefits	Resistance issues
Signaling Pathway Inhibitors, e.g., MEK Inhibitors	Cell signaling pathways	Under investigation	Needs to be combined with platinum drugs to enhance efficacy
Tumor Vaccines	Specific antigens	Under investigation	Immunosuppressive factors in the tumor microenvironment affect vaccine efficacy
Cell Therapy, e.g., CAR-T Cell Therapy	Tumor cells	Under investigation	Immune evasion mechanisms in the tumor microenvironment
Epigenetic Therapy	DNA methylation, histone modifications	Preclinical studies	Off-target effects may impact efficacy
Oncolytic Virus Therapy	Tumor cells	Under investigation	Needs to be combined with immune checkpoint inhibitors to enhance efficacy

HGSOC, High-Grade Serous Ovarian Cancer; PARP, Poly (ADP-ribose) Polymerase; VEGF, Vascular Endothelial Growth Factor; MEK, Mitogen-Activated Protein Kinase; CAR-T, Chimeric Antigen Receptor T-cell.

## 6 Clinical translation and future directions

Multi-omics integrated analysis emerges as an innovative research approach, enabling an in-depth exploration of HGSOC drug resistance mechanisms across genomic, transcriptomic, and proteomic levels. By integrating data from different omics platforms, researchers can uncover biomarkers related to drug resistance and signaling pathways.

In HGSOC drug resistance research, integrating multiple omics datasets is particularly crucial. First, high-throughput sequencing technologies generate genomic and transcriptomic data, which, when combined with bioinformatics analysis, can identify mutations and expression changes associated with drug resistance (147). Additionally, proteomics applications can further validate the functional and clinical relevance of these molecular markers (109). By integrating these datasets, researchers can construct network models of the resistance mechanisms. For instance, using CRISPR-Cas9 technology to functionally validate specific genes can help confirm their roles in the drug resistance process (48). This multi-level integrated analysis not only enhances the understanding of resistance mechanisms but also provides theoretical foundations for personalized treatment strategies.

Integrated analysis of HGSOC drug resistance demonstrates broad prospects for clinical applications. By identifying drug resistance biomarkers, physicians can select treatment protocols more precisely, thereby improving patient survival rates and quality of life. Studies have shown that combining the ratios of CA125 and adiponectin levels can effectively predict patient resistance to platinum drugs, offering potential biomarkers for early resistance identification (148).

The application of big data is particularly critical in personalized HGSOC treatment. By analyzing patient genomic data and clinical characteristics, researchers can construct models for predicting drug resistance (149). These models can help physicians select the most appropriate treatment protocols and dynamically adjust them during the treatment process. For example, the DRDscore, based on multi-omics data, can effectively predict platinum drug resistance and guide clinical treatment (150).

The emergence of nanomedicine delivery systems offers new hope for overcoming drug resistance in HGSOC. These systems modify the physicochemical properties of drugs, increasing drug concentration at tumor sites and enhancing therapeutic effects (151). In HGSOC treatment, the types of nanocarriers are diverse, including liposomes, polymer nanoparticles, and inorganic nanoparticles, each with unique functions and applications (15, 152).

Nanocarriers can achieve selective drug release through both passive and active targeting mechanisms. Utilizing characteristics of the tumor microenvironment, such as acidic environments or excess glutathione, can significantly enhance drug accumulation in tumor cells while reducing toxicity to normal cells (153). Liposomes can effectively encapsulate water-soluble drugs, improving their pharmacokinetic properties, while polymer nanoparticles can achieve tumor-targeted delivery by modifying their surface properties (154). Research indicates that certain nanocarriers can enhance tumor cell endocytosis, improving cellular uptake of drugs and overcoming resistance (155). Designing and optimizing these nanocarriers is a current research focus aimed at improving treatment outcomes in HGSOC.

The emergence of CRISPR-Cas9 technology provides powerful tools for investigating the mechanisms of HGSOC drug resistance. Through gene editing, researchers can precisely knock out or activate genes associated with resistance, revealing their roles in HGSOC drug resistance. For instance, studies have discovered that certain anti-apoptotic genes are upregulated in HGSOC cells and are closely associated with chemotherapy resistance (156). Using CRISPR-Cas9 to edit these genes can help researchers better understand resistance mechanisms and provide a foundation for developing new treatment strategies.

Future research must focus on several key areas to address drug resistance in HGSOC. First, an in-depth investigation of the tumor microenvironment’s impact on resistance, particularly the interactions between immune and tumor cells, may provide insights for developing novel immunotherapies. Second, targeted therapies that address specific gene mutations or epigenetic changes will become crucial research directions, such as exploring the relationships between BRCA mutations and PARP inhibitor resistance. Finally, emerging technologies, such as single-cell sequencing and liquid biopsy, can provide more precise biomarkers for personalized treatment, improving therapeutic outcomes and reducing the risks of resistance.

## 7 Conclusion

This review summarizes the latest advances in drug resistance mechanisms and the clinical translation of High-Grade Serous Ovarian Carcinoma (HGSOC). As the most lethal subtype of ovarian cancer, the drug resistance mechanisms of HGSOC present complex and diverse characteristics, primarily involving tumor cell heterogeneity, dynamic microenvironment changes, and abnormal expression of specific molecular markers. Research indicates that the drug resistance mechanisms of HGSOC are closely associated not only with abnormal intracellular signaling pathways but also with complex interactions between fibroblasts and immune cells in the tumor microenvironment.

Specifically, the drug resistance mechanisms of HGSOC are primarily manifested in tumor cell heterogeneity, which leads to significant differences in drug response among various cell subpopulations. The existence of cisplatin-resistant cell subpopulations (E0) significantly impacts patient prognosis. Moreover, the close association between key molecular expressions, such as EZH2, and drug resistance further highlights their critical roles in these resistance mechanisms. Tumor microenvironment remodeling and hypoxic conditions are also considered key factors that influence drug resistance. The application of emerging technologies, such as single-cell RNA sequencing and liquid biopsy, in recent years has provided new biomarkers for monitoring HGSOC drug resistance, helping to identify resistant cell subpopulations and offering important foundations for personalized treatment.

In terms of treatment strategies, although existing chemotherapy protocols are somewhat effective, they still face limitations, such as drug resistance and side effects. Therefore, a comprehensive evaluation combining targeted therapy, immunotherapy, and emerging therapies can significantly improve the survival rates and quality of life of HGSOC patients. Research on HGSOC drug resistance not only provides crucial insights into tumor biology but also establishes a solid foundation for developing future treatment strategies. By delving into resistance mechanisms, researchers can identify new therapeutic targets and promote the development of novel treatment strategies.

The clinical significance of research on HGSOC drug resistance lies in providing scientific evidence for personalized treatment. By thoroughly understanding resistance mechanisms, clinicians can develop more precise treatment plans based on patients’ specific molecular characteristics, thereby significantly improving treatment outcomes. In-depth research on resistance will drive the development of new therapies, especially in the domains of targeted and immunotherapy. By identifying molecular markers associated with drug resistance, researchers can design more targeted treatment strategies to overcome existing limitations in treatment. However, the aforementioned HGSOC resistance mechanisms, and relevant biomarkers or drugs targeting reversing HGSOC drug resistance should be verified in future large patient cohort studies to examine efficiency and safety.

In HGSOC research, interdisciplinary collaboration is particularly important. Experts from biomedicine, molecular biology, and clinical medicine should work together to promote the close integration of basic research and clinical applications, ultimately achieving more effective treatment protocols. Future research should focus on advancing personalized treatment and precision medicine. By utilizing emerging technologies, such as single-cell sequencing and liquid biopsy, researchers can more accurately identify resistance mechanisms and biomarkers, providing HGSOC patients with more precise treatment plans and ultimately improving their prognosis and quality of life. In addition, future studies should focus more on addressing the multi-drug resistance (MDR) mechanisms and utilizing the cell or gene therapies to overcome MDR in cancer cells, which paves the way for the precision and personalized medicine for patients.
